# Late is not great: fitness implications of delayed symbiont acquisition

**DOI:** 10.3389/fmicb.2026.1786420

**Published:** 2026-05-14

**Authors:** Liam T. Sullivan, Suzanne E. Kelly, Martha S. Hunter

**Affiliations:** 1 Graduate Interdisciplinary Program in Entomology and Insect Science, University of Arizona, Tucson, AZ, United States; 2 Department of Entomology, University of Arizona, Tucson, AZ, United States

**Keywords:** *Caballeronia*, developmental symbiosis, gut morphogenesis, *Leptoglossus zonatus*, symbiont transmission

## Abstract

Acquisition of beneficial microbes is often vital to the success of animals. In terrestrial arthropods, obligate symbionts are often acquired from mothers. However, the leaffooted bug, *Leptoglossus zonatus*, acquires its soil-dwelling bacterial symbiont, *Caballeronia*, from the environment in each generation starting in the 2nd instar. Bugs must locate their symbionts in a complex and heterogeneous environment, and location and acquisition may take time. We asked if there are costs to acquisition delay and if such costs accrue over longer intervals. A cohort of *L. zonatus* nymphs was split among eight treatments: seven different intervals for symbiont acquisition after bugs reached the 2nd instar (0–24 days) and a negative control in which bugs were never fed *Caballeronia*. We assessed the impacts on host performance by measuring acquisition success, juvenile survivorship, development time, adult mass, and development of the midgut symbiotic organ compared with the 0-day treatment. All measures were reduced by delayed acquisition of symbionts, beginning 8 days after nymphs reached the 2nd instar. As the delay increased, host survivorship and acquisition success were further reduced. Surviving hosts developed more slowly, were smaller, and their midgut symbiotic organs were smaller. Our results suggest that symbiont acquisition is most beneficial to hosts within a narrow window, within ~1 week of reaching the 2nd instar. Our results point to an important potential cost to this type of symbiosis. Nymphs must not only find the symbiont, but find it before it is too late to provide a benefit.

## Introduction

Symbiotic microbes can provide essential services that facilitate and promote the growth and development of their animal hosts (Pradeu, 2011; Carrier and Bosch, 2022). For many hosts, the presence of the beneficial associate is necessary for survival. In insects, beneficial symbionts often confer nutritional benefits, acting to digest complex molecules such as lignin or pectin (Ahmad et al., 2022; Kirsch et al., 2025), to synthesize essential nutrients from dietary building blocks, and to recycle limited nitrogen from waste (Kaltenpoth et al., 2025). Due to these benefits, microbial partners may have facilitated an evolutionary expansion of the host niche, allowing insects to utilize new dietary resources (Hansen and Moran, 2014). While the functional roles of symbionts vary greatly, all of these partnerships must be sustained across generations. Symbiont transmission route varies and can be ordered on a spectrum, from vertical (parent to offspring; Wilkinson et al., 2003) to horizontal (from conspecific sources) to the environmental acquisition of free-living microbes. The latter has also been categorized as a kind of horizontal transmission (Bright and Bulgheresi, 2010; Salem et al., 2015). While vertically transmitted symbionts are maintained intra- or extracellularly in hosts between generations, horizontal and environmentally-acquired partners are shared between individuals through oral or anal trophallaxis or fecal consumption (coprophagy) or found within the host’s habitat.

Each transmission mode comes with costs and benefits. Vertical transmission efficiency is often nearly perfect (Hosokawa et al., 2013; Ballinger and Perlman, 2017) and such long-term associations may span millions of years (Moran et al., 2005). However, the bottleneck of vertical transmission often leads to degradation of the symbiont genome, with symbionts generally maintaining only essential metabolic functions for themselves and their hosts (McCutcheon and Moran, 2012; Moran and Bennett, 2014). As a consequence, many hosts and symbionts become irreversibly integrated, limiting a host to a single partner or partner strain. With this restriction, host fitness may be constrained should the environmental conditions change, causing the symbiosis to be conditionally deleterious (Zhang et al., 2019; Bruijning et al., 2021). In contrast, some hosts acquire their symbiont from wild microbial communities in the diet, soil or water (Bright and Bulgheresi, 2010; Kikuchi et al., 2011a; Salem et al., 2015). To persist in harsh environmental conditions and compete within free-living microbial communities, these symbionts must retain a large genome, robust metabolic capabilities and be able to adapt to the external, local environment. The relatively large genetic toolkit of these microbes has the potential to provide novel, locally adapted benefits to a host that acquires them (Kikuchi et al., 2012; Tago et al., 2015). Environmental partners, however, also likely come with costs. Acquisition of a partner from the environment may pose a greater risk to the host of acquiring less beneficial or detrimental partners that have not yet been vetted by rounds of selection. Additionally, an even greater challenge for hosts with environmental partners may be locating the symbiont at all, and in time for it to be useful to the host.

The arboreal leaffooted bug *Leptoglossus zonatus* Dallas (Hemiptera: Coreidae) belongs to one of at least nine families of true bugs within the Pentatomomorpha associated with betaproteobacteria in the genus *Caballeronia* formerly *Burkholderia*; (Kikuchi et al., 2011a; Sudakaran et al., 2015; Lirette et al., 2025). This symbiosis was first described in the model host for this system, *Riptortis pedestris* (Hemiptera: Alydidae) by Kikuchi et al. (2007). Nymphs acquire their symbiotic partner from the environment as juveniles during the 2nd instar. *Caballeronia* are free-living, widely distributed soil microbes (King and Parke, 1996; Tabacchioni et al., 2002; Ramette et al., 2005) that, when ingested by a bug of this group, colonize the midgut 4 region (M4) and induce development and elongation of the midgut crypts (Kikuchi et al., 2007, 2011a; Takeshita and Kikuchi, 2017; Jang et al., 2023). Once colonized, *Caballeronia* serve several functions. The growing bacterial population is an important nutritional source, synthesizing essential amino acids and B vitamins (Kim et al., 2013; Ohbayashi et al., 2019). In addition, the presence of the *Caballeronia* upregulates the host immune system (Jang et al., 2024) and host development by increasing the production of hexamerin and vitellogenin (Lee et al., 2017).

The bug—*Caballeronia* relationship, akin to the *Vibrio*—squid association (Nyholm and McFall-Ngai, 2004), is highly specialized, with nymphs selectively acquiring symbionts from the complex soil environment (Lextrait et al., 2025). *Caballeronia* and other closely related members of the *Burkholderia sensu lato* are filtered through a constricted region (CR) upstream of the M4. The CR restricts access to the M4 so that only *Caballeronia* and some closely related species can enter; colonizing microbes wrap their flagella around their cells and twist forward like a corkscrew to traverse into the M4 crypts (Kinosita et al., 2018; Junker et al., 2025). The reliance upon *Caballeronia* varies among host species, but it is strictly obligate for *L. zonatus* (Hunter et al., 2022). Individuals that fail to acquire the symbiont exhibit increased development time, reduced adult mass, and do not reproduce (Hunter et al., 2022; Umanzor et al., 2025).

While a failure to acquire the symbiont is catastrophic for the host in this system, the risk of a delay in symbiont acquisition is unknown. Delay is likely to have impacts on development and host performance generally (Kikuchi et al., 2011b). *Caballeronia* are cosmopolitan but comprise a small percentage of the larger soil community (King and Parke, 1996; Tabacchioni et al., 2002; Ramette et al., 2005) and appear to be sensitive to soil alkalinity (Stopnisek et al., 2014; Itoh et al., 2025) and aridity (Parajuli et al., 2025). It could be that hemipteran hosts select habitat with *Caballeronia* nearby, so species that dwell near the soil are assured of a nearby symbiont. However, many coreids, including *L. zonatus,* are largely arboreal, with nymphs emerging from eggs high above the soil in tree canopies (Fernandes et al., 2015). Within 3–4 days, nymphs reach the 2nd instar and become receptive to colonization by *Caballeronia*. Recently we showed that individuals rarely acquire *Caballeronia* when they are confined to foliage in the canopy (Sullivan et al., 2025), suggesting that the symbiont is not common on tree leaf surfaces and that nymphs may need to disperse or to wait for the symbiont to become available. Soil is likely the main repository of the symbiont, although conspecific frass may sometimes be an important intermediary for these gregarious insects (Villa et al., 2023; Sullivan, unpublished data). We hypothesize that there is a finite window for *Caballeronia* acquisition for *L. zonatus,* within which normal development occurs with minimal consequence to host fitness. We also hypothesize that outside this window, longer intervals before symbiont acquisition cause greater reductions in insect performance.

In the current study, we explored the impact of delayed symbiont acquisition on *L. zonatus* performance, including development time from nymph to adulthood, juvenile survivorship, and mass at eclosion. To address this, a single cohort of *L. zonatus* was reared and fed *Caballeronia* at one of seven time points (0, 4, 8, 12, 16, 20, or 24 days) after reaching the second instar, or in an additional negative control, bugs remained aposymbiotic. Given the induction of development of the gut by symbiont colonization in *R. pedestris,* we also asked whether the colonization of the M4 symbiotic organ was time-limited in *L. zonatus* and whether gut morphogenesis, and particularly M4 crypt growth, was influenced by the timing of symbiont acquisition.

## Methods

### Insect rearing and symbiont feeding

*Leptoglossus zonatus* were reared in a colony at the University of Arizona in Tucson, AZ, USA (Hunter et al., 2022). Insects were reared and the experiment performed at 27 °C at 16:8 L/D. Cultures were maintained on potted cowpea plants (*Vigna unguiculata*) in cages with raw shelled peanuts provided as food. Eggs were collected from the cages at 24 h intervals from dozens of mothers to produce a cohort of more than 500 individuals that hatched within 24 h.

### Experimental insect rearing

Eggs and first instar nymphs were held in Petri dishes with 2 mL vials of deionized water with 0.05% ascorbic acid (DWA). Upon reaching the 2nd instar, nymphs were sorted into boxes, mixing broods from Petri dishes as much as possible to minimize bias due to clutch. Boxes were plexiglass (11.3 cm × 11.3 cm × 4 cm) with a fabric mesh square in the lid. Prior to use, boxes were washed thoroughly and soaked in 0.1% bleach solution. Boxes were provisioned with washed cowpea (*Vigna unguiculata*) seedlings embedded in 0.4% water agar in floral water vials. Boxes also contained raw shelled peanuts and deionized water in 2 mL glass vials. After the boxes were each allotted eight 2nd instar nymphs, they were randomly assigned to one of the eight treatments. Each treatment was replicated in eight boxes.

### Experimental treatments

Treatments were assigned based on when *Caballeronia* was presented to nymphs. Treatments were defined by the number of days following the start of the 2nd instar. We chose days rather than developmental stage because of the highly variable development time of aposymbiotic individuals and the potential for bias in treatment assignment because of that variation (Hunter et al., 2022). The treatments were as follows: zero-day treatment (hereafter “0 day”), in which nymphs were presented *Caballeronia* on the day of their molt to the second instar; a negative control, in which nymphs were never presented with the symbiont; and delays of symbiont presentation that were 4, 8, 12, 16, 20, and 24 days after the second instar molt. Development and mortality of bugs in each box were monitored three times weekly for the duration of the experiment. At each assessment, dead nymphs were removed, and peanuts, water, and seedlings were replaced as necessary. As individuals reached the 5th instar, boxes were placed on their edge to increase the depth of the enclosure and facilitate eclosion of adults. When adults eclosed, they were removed from the box and their fresh mass measured. Adults were stored individually at −80 °C for later dissection and assessment of symbiotic status.

### Symbiont culturing and feeding

Yeast-glucose (YG) agar plates were streaked from glycerol freezer stocks of green fluorescent protein (GFP) labeled *Caballeronia* (strain Lz049G; Sullivan et al., 2025) and grown at room temperature for several days. A single colony was used to inoculate liquid YG media and grown at room temperature overnight. Multiple tubes of fresh broth were then inoculated with 100 μL of the overnight culture and grown until log phase was reached (0.8 OD). Freezer stock aliquots to be used for feedings were made by mixing this culture 50:50 with 80% glycerol and stored at -80 °C.

For feedings, the frozen aliquots were thawed, spun down, resuspended in sterile deionized water, and diluted to 0.06 OD in sterile DWA for presentation to the insects. Prior to symbiont presentation, water was withheld for 4–6 h to encourage nymph feeding. The *Caballeronia* suspension was presented to the bugs in 25 mm Petri dishes containing cotton gauze saturated with ~1.5 mL of bacterial suspension. Symbiont feedings occurred twice during a 48-h period, to promote acquisition.

### Dissection, microscopy, gut measurements

To assess symbiotic status, the entire length of the adult midgut 4 (M4) region was removed and stretched to full length on glass slides. These gut sections were wet-mounted in sterile 1× phosphate-buffered saline (PBS) and held in humidification chambers until imaging was completed. For imaging, the M4 tissue was gently flattened using glass coverslips. The full length of the M4 was examined with a fluorescence microscope at 20x magnification under both bright field and fluorescent settings to determine the presence of GFP *Caballeronia*. Following imaging, the M4 gut sections were stored in 100% EtOH at −20 °C.

Fluorescence microscopy to detect the GFP-labeled symbiont was performed using an Olympus BX53 microscope with a white light source, X-cite 120LED Boost illumination system and Brightline GFP-3035D-000 filter, composed of 472 and 520 nm filters and 495 nm single-edge dichromatic beamsplitter (Semrock, Inc.). Images were captured using an Olympus DP74 camera and processed with Olympus cellSens Standard 1.6 software. Image analysis was performed with Fiji, a distribution of ImageJ2 (Schindelin et al., 2012).

Four or more adults from each “delay” treatment that had been presented with GFP-*Caballeronia* and showed fluorescence were selected at random to determine if the M4 crypt size was affected by the timing of symbiont acquisition. Individuals from the negative control were included for comparison of gut development in the absence of the symbiont. Crypt measurements were performed using Fiji, a distribution of ImageJ2. For most individuals, 10 crypts from across the length of the M4 were measured to capture the variability in crypt size. Crypt length and width were assessed with the line tool, measuring from the crypt aperture to apex or from epithelial wall to wall, respectively. Crypt area was measured using the spline tool following the perimeter of the crypt. No fluorescence was observed from bugs in the 24-day treatment, therefore all adults in this treatment were aposymbiotic (lacked symbionts) and no gut measurements were recorded.

### DNA extraction, diagnostic PCR

Following imaging, bugs that were GFP-negative underwent diagnostic PCR to check for potential contaminant *Caballeronia* or related Burkholderiaceae. Prior to DNA extraction, the M4 tissue was transferred to sterile, deionized water for at least 5 min to remove ethanol from the tissue and then was placed into 0.5 mL tubes of 5–10% Chelex 100 (MilliporeSigma) solution and ground using a pipette tip. Eight microliter of proteinase K (20 mg/mL) was added to each tube, and incubated overnight at 37 °C, heated to 96 °C, and stored at −20 °C until needed.

Diagnostic PCR was performed using primers for *Burkholderia sensu lato* specific 16S rRNA Burk16SF (5′-TTTTGGACAATGGG GGCAAC-3′) and Burk16SR (5′-GCTCTTGCGTAGCAACTAAG-3′). We note that these primers amplify *Burkholderia* and relatives other than *Caballeronia* that can colonize the M4 (Itoh et al., 2019). Thermocycler conditions were set to: 95 °C for 10 min, 30 cycles of 95 °C for 30s, 55 °C for 1 min and 72 °C for 1 min (Kikuchi et al., 2005). PCR products were visualized on an agarose gel with SYBR Green (Hunter et al., 2022).

There were relatively few bugs found to be GFP negative but PCR positive for *Burkholderia sensu lato*: 15 of 266 adults were GFP-negative and PCR positive across the “delay” treatments, and none were GFP negative and PCR positive in the negative control. These few contaminated bugs in the delay treatments were excluded from all analyses of adults because the colonization by environmental *Caballeronia* or related bacteria had occurred at an unknown interval since the 2nd instar.

*Statistical Analyses.* All analyses were performed in R version 4.5.0, (R Core Team, 2025) and RStudio (Posit team, 2024). Figures were generated with ggplot2 (Wickham, 2016).

### Performance

Bug performance was measured by symbiont acquisition success, survivorship, development time, mass, and midgut morphogenesis. For each measure, unless specified below, contrasts for the estimated marginal means were made between each treatment and the 0-day treatment, and between each treatment and the negative control, then extracted using *emmeans* (Lenth, 2025) when appropriate.

Survivorship was calculated as the proportion of individuals that survived to adulthood within each replicate. Initially, these data were analyzed with a binomial generalized linear mixed model with replicate as a random effect. However, the inclusion of replicate resulted in singularity of fit. The effect of treatment on survivorship was instead assessed using a generalized linear model with a quasibinomial distribution to accommodate overdispersion (*lme4*; Bates et al., 2015). Model selection was performed using *drop1.* Similarly, acquisition success was described as the proportion of GFP positive adults within each treatment. Due to complete separation and overdispersion in the data, Firth’s penalized logistic regression was used with a quasibinomial estimation (*logistf*; Heinze et al., 2025).

Development time was calculated as the number of days from the start of the second instar until the beginning of the adult stage. Differences in development time were assessed using a generalized linear mixed model under a quasipoisson estimation (*lme4*; Bates et al., 2015) to accommodate underdispersion in the data. Treatment served as a fixed effect and replicate number was a random effect. Due to singularity of fit, the random effect was removed, and the analysis simplified to a generalized linear regression with a quasipoisson estimation.

Adult mass was assessed using a generalized linear model with treatment and sex as predictors *(lme4).* Replicate was included in the initial mixed model but was removed due to singular fit. Sex was included because *L. zonatus* males are smaller than females (Hunter et al., 2022). Midgut 4 crypt measurements were assessed using a linear mixed model with insect as a random effect (*lmer4*), as multiple crypt measurements were made for each individual bug sampled.

## Results

### Acquisition success

**Figure 1:**
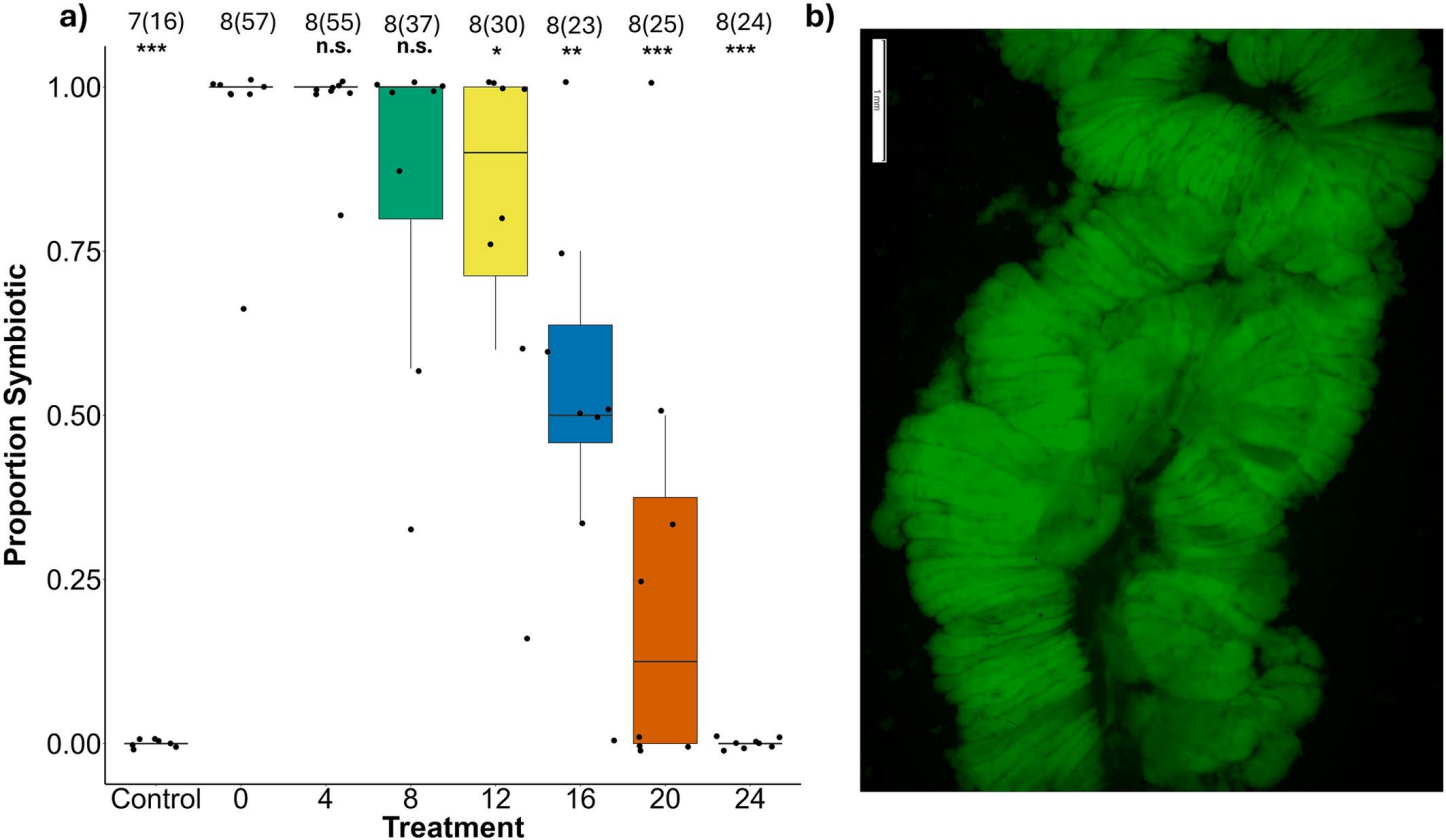
**(a)** Proportion of *L. zonatus* adults that were colonized with GFP-labeled *Caballeronia* within treatment group (days after reaching the 2nd instar that nymphs were provided with *Caballeronia).* The numbers above the boxes are the number of replicates and within parentheses are the number of individual insects measured. Asterisks represent the *p* values when each treatment was compared with the 0 day treatment (0; n.s. = non-significant, * ≤ 0.05, ** ≤ 0.01, *** ≤ 0.001). **(b)** Example of GFP-labeled *Caballeronia* occupying the crypts of a section of the M4 symbiotic organ. Scale bars represent 1 mm. Photograph credit: C. J. Terrell.

Symbiont acquisition success was reduced with increasing delays before symbiont exposure (LRT = 130.48, *p* < 0.0001). While a 4 day delay resulted in equivalent rates of *Caballeronia* acquisition relative to the 0 day treatment (*z* = 0.210, *p* = 0.997) with 98 ± 0.13% and 96 ± 0.12% of individuals acquiring *Caballeronia,* respectively, acquisition success declined significantly beginning 8 days after the beginning of the 2nd instar (8 days: *z* = −3.978, *p =* 0.001; 12 days: *z* = −4.43, *p* = 3.06 × 10^−4^; 16 days: *z* = −5.49*, p* = 8.21 × 10^−6^; 20 days: *t* = −5.00 6, *p* = 4.58 × 10^−5^). By 24 days, none of the few bugs alive were able to acquire the symbiont in the M4 (24 days: *z* = −3.93, *p* = 0.002) (Figure 1; Supplementary Tables 1, 2). Negative control bugs were never offered the symbiont so are not included in this analysis.

### Survival to adulthood

**Figure 2:**
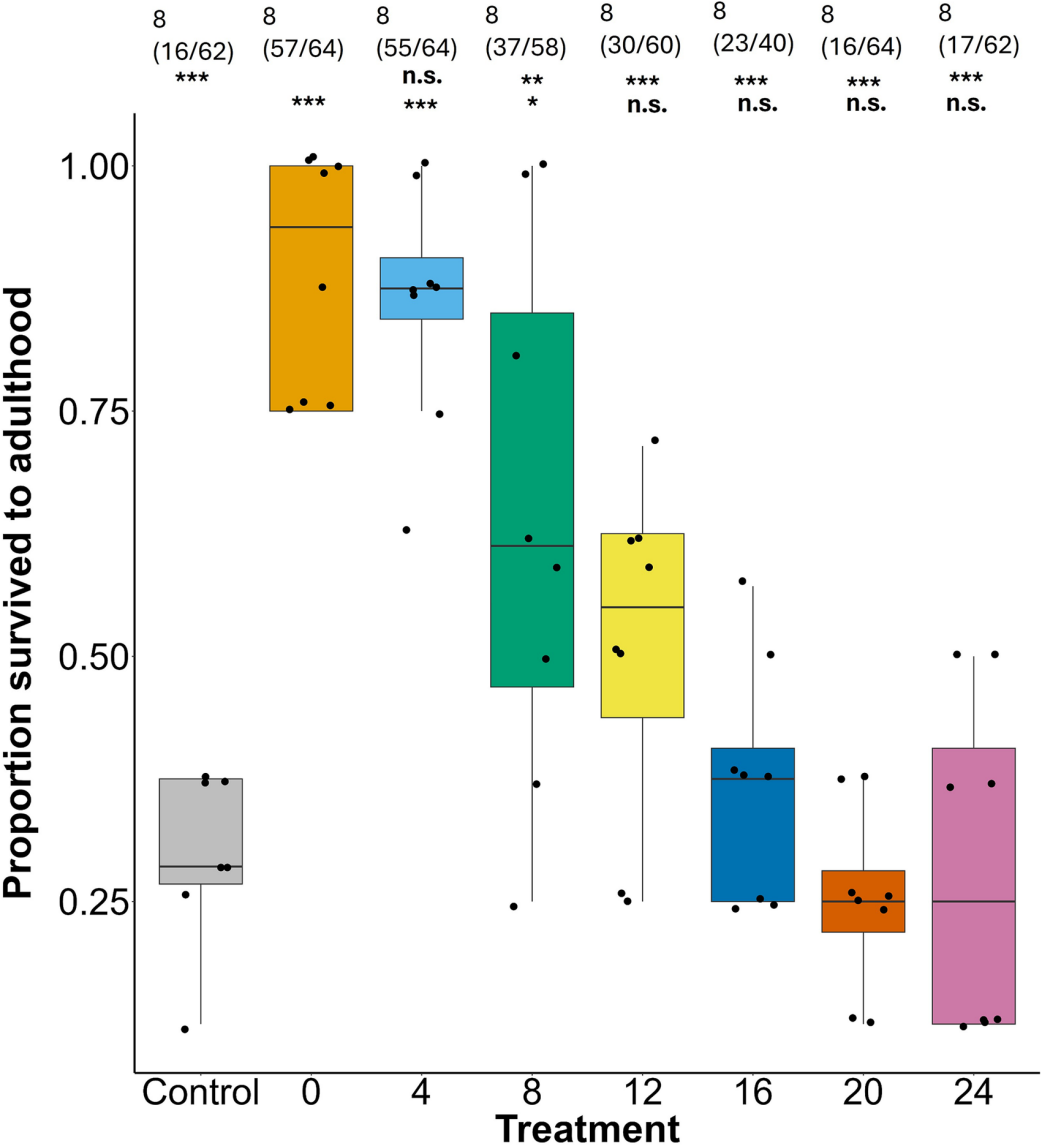
Proportion of *L. zonatus* nymphs that survived to adulthood in different treatment groups (days after reaching the 2nd instar that nymphs were provided with *Caballeronia*). The numbers above the boxes are the number of replicates and within parentheses are the number of individual insects measured. The upper row of asterisks represents the *p*-values when each treatment was compared with the 0 day treatment (0), the lower row represents comparisons with the negative control (control; n.s. = non-significant, * ≤ 0.05, ** ≤ 0.01, *** ≤ 0.001).

A delay in access to the symbiont also dramatically reduced survival to adulthood (LRT *=* 135.35, *p* < 0.0001). Nymphs in the 4 day treatment were as likely to survive as those in the 0-day treatment (4 days: *z* = −0.533, *p* = 0.969), with 85.94% of nymphs reaching adulthood compared to 89.06% in the 0 day treatment. Survival declined significantly in the 8 day treatment (8 days: *z* = −3.16, *p =* 0.010) with only 63.79% of nymphs reaching adulthood. Bugs in all treatments with longer delays showed stronger reductions in juvenile survivorship compared to the 0-day treatment (12 days: *z* = −4.40, *p* = 7.42 × 10^−5^; 16 days: *z* = −5.40*, p* = 2.11 × 10^−7^; 20 days: *z* = −6.47, *p* = 6.70 × 10^−10^; 24 days: *z* = −6.25, *p* = 2.88 × 10^−9^) (Figure 2; Supplementary Tables 3, 4). The aposymbiotic negative control was the most affected, with only 28.57% of nymphs surviving to adulthood (*z* = −6.37, *p* = 1.34 × 10^−9^). When compared with the aposymbiotic negative control, survival was significantly higher in the 0, 4, 8, and 12 day treatments, but was not significantly different from the 16, 20, and 24 day treatments (Figure 2; Supplementary Table 5).

### Insect development

**Figure 3:**
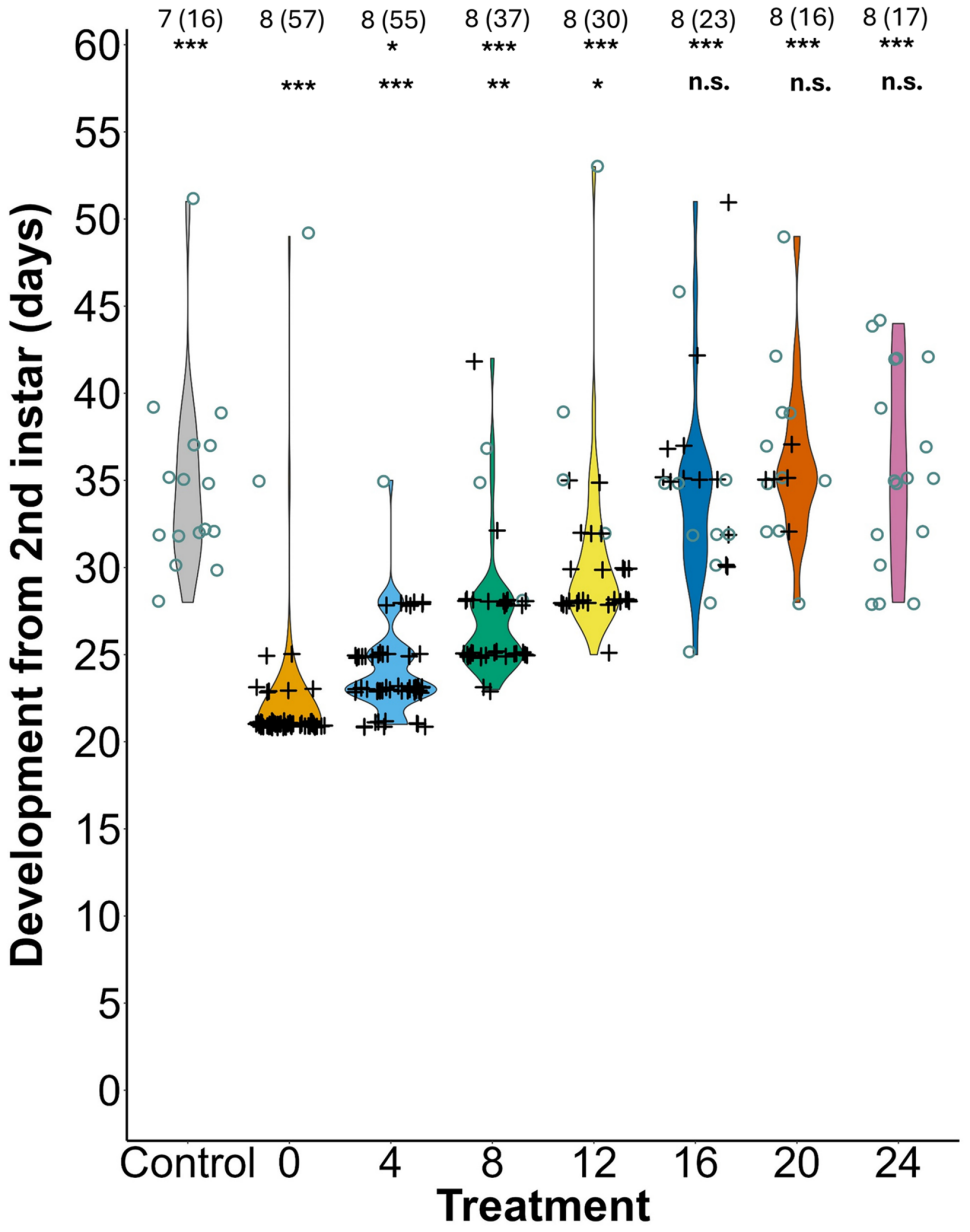
Development time of *L. zonatus* from 2nd instar to adult in treatments that varied by the number of days after reaching the 2nd instar that nymphs were provided with *Caballeronia.* The numbers above the plots are the number of replicates and within parentheses are the number of individual insects measured. Symbiotic status is represent by with “O” (GFP-negative) or “+” (GFP-positive). The upper row of asterisks represent the *p* values when each treatment was compared with the 0 day treatment (0), the lower row represents comparisons with the negative control (n.s. = non-significant, * ≤ 0.05, ** ≤ 0.01, *** ≤ 0.001).

The time *L. zonatus* took to develop to adulthood was significantly affected by the timing of symbiont access (*F* = 59.01, *p* < 0.0001). The shortest development time was observed when nymphs received the symbiont on the day they reached the second instar (0 day treatment) followed closely by the 4 day treatment (*z =* 2.64*, p* = 0.048), with bugs reaching adulthood in 22.1 ± 4.16 and 24 ± 2.55 days, respectively. Development time increased significantly for individuals that were delayed 8 days in acquiring the symbiont (27.2 ± 3.84 days, *z =* 6.16, *p* = 5.10 × 10^−9^) and for each treatment with a delay longer than 8 days (12 days: *z* = 9.25, *p* = 3.97 × 10^−14^; 16 days: *z* = 12.23*, p* < 0.0001; 20 days: *z* = 11.89, *p* < 0.0001; 24 days: *z* = 12.05, *p* < 0.0001) (Figure 3; Supplementary Tables 6, 7). The few surviving insects in the aposymbiotic negative control took 57% longer to develop compared to the 0-day treatment (34.8 ± 5.41 days, *z* = 11.00, *p* = 2.86 × 10^−14^). When compared to the aposymbiotic negative control group development the 0, 4, 8, and 12 day treatments developed significantly faster, while development times for the 16, 20, and 24 days treatments were not significantly different (Figure 3; Supplementary Table 8).

**Figure 4:**
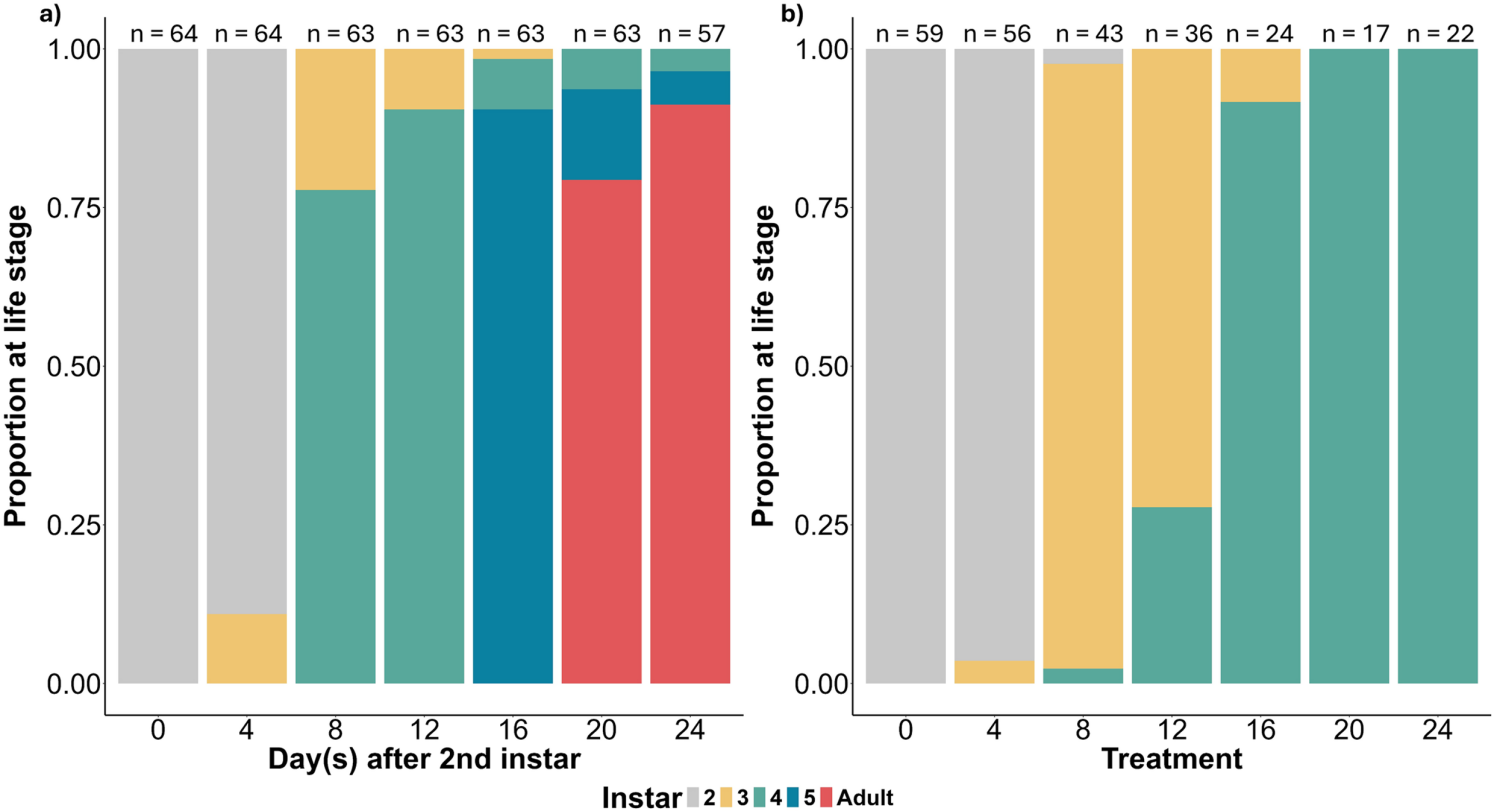
**(a)** Estimated proportion of individuals in the 0 day treatment at each life stage in days after the start of the 2^nd^ instar nymph molt. **(b)** Estimated proportions of the life stages of bugs on the day they were given access to *Caballeronia*. The delay in life stage in this figure relative to the 0-day treatment bugs in this figure is due to the aposymbiotic state of bugs on their treatment day and the slower development time of these individuals. This dataset includes a small number of contaminated samples (15 of 266 total samples) that could not be removed due to data collection methods.

The slower development time observed in the aposymbiotic and later delay treatments resulted in a long extension of the third and fourth instar stages relative to the 0-day treatment nymphs (Figures 4a,b). For the treatments in which most measures of performance declined significantly, starting in the 8 or 12 day delay treatments, most aposymbiotic nymphs had graduated to the third instar nymphal stage (Figure 4b). By 16 or 20 days, when performance declined even more precipitously, most nymphs had graduated to the fourth instar (Figure 4b).

### Adult mass

**Figure 5:**
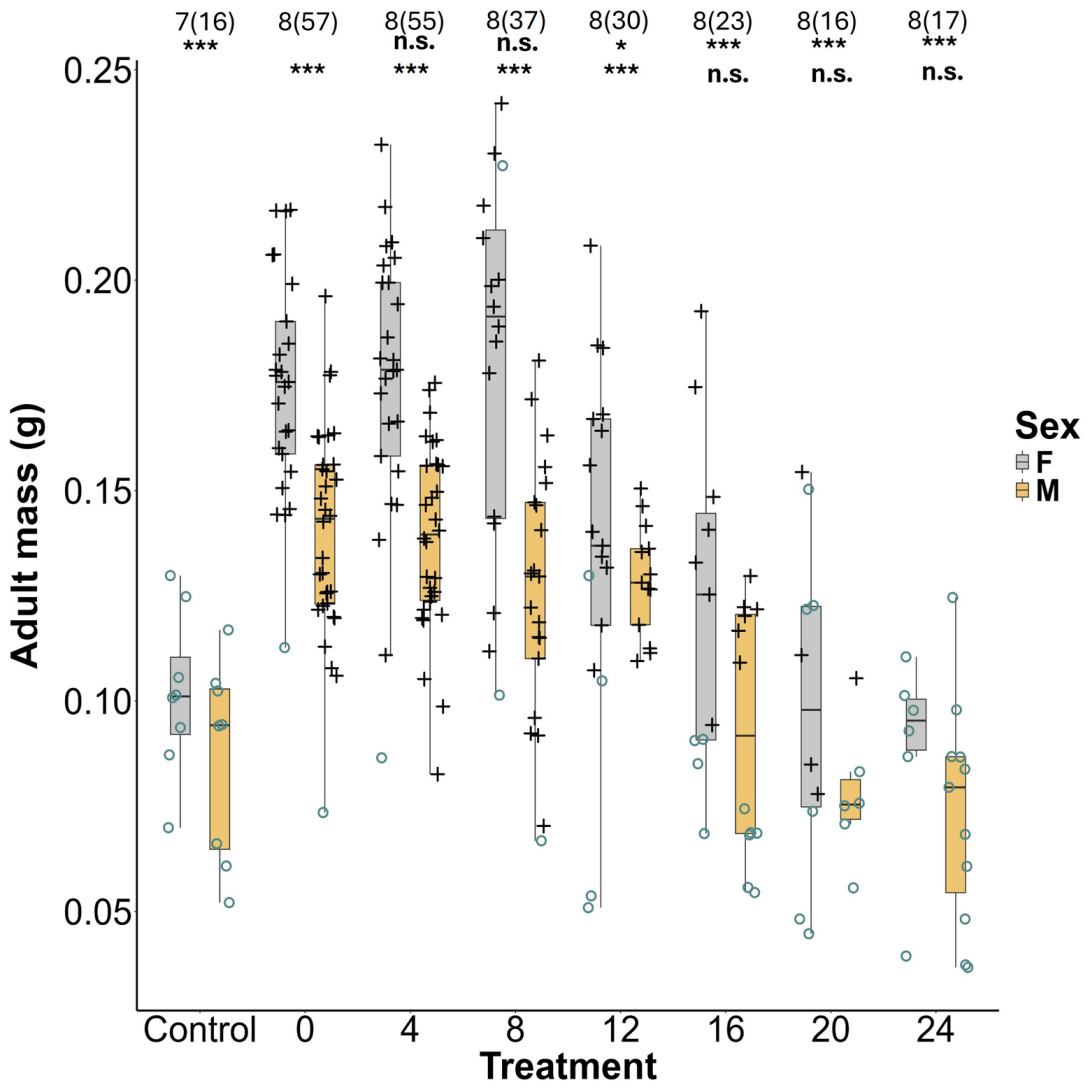
Fresh mass of *L. zonatus* females and males by treatment group (days after reaching the 2nd instar that nymphs were provided with *Caballeronia*). The numbers above the bars are the number of replicates and within parentheses are the number of individual insects measured. Symbiotic status is represent by with “O” (GFP-negative) or “+” (GFP-positive). The upper row of asterisks represent the *p* values when each treatment was compared with the 0 day treatment, the lower row represents treatment comparisons with the negative control (Control) (n.s. = non-significant, * ≤ 0.05, ** ≤ 0.01, *** ≤ 0.001).

Adult mass varied significantly with sex, with females being 1.23 times the mass of males on average (LRT = 59.18, *p* = 1.44 × 10^−14^). Mass was also significantly influenced by symbiont delay (LRT = 149.60, *p* < 2.2 × 10^−16^). Mass declined significantly from the 0 day treatment (0.155 ± 0.031 g) beginning in the 12-day delay treatment (0.134 ± 0.033 g; *t* = −3.72, *p* = 0.002; 16 days: *t* = −6.62*, p* = 1.61 × 10^−9^; 20 days: *t* = −8.10, *P* 3.05 × 10^−13^; 24 days: *t* = −8.75, *p* = 1.14 × 10^−13^) (Figure 5; Supplementary Tables 9, 10). When compared with the 0-day treatment, adult mass was almost 40% less in the aposymbiotic negative control (0.094 ± 0.22 g; *t* = −7.31, *p* = 2.53 × 10^−11^). When compared to the negative control group, insects in the 0, 4, 8, and 12 day treatments were significantly heavier, while the 16, 20, and 24 days treatments were not significantly different (Figure 5; Supplementary Table 11).

### Midgut development

**Figure 6:**
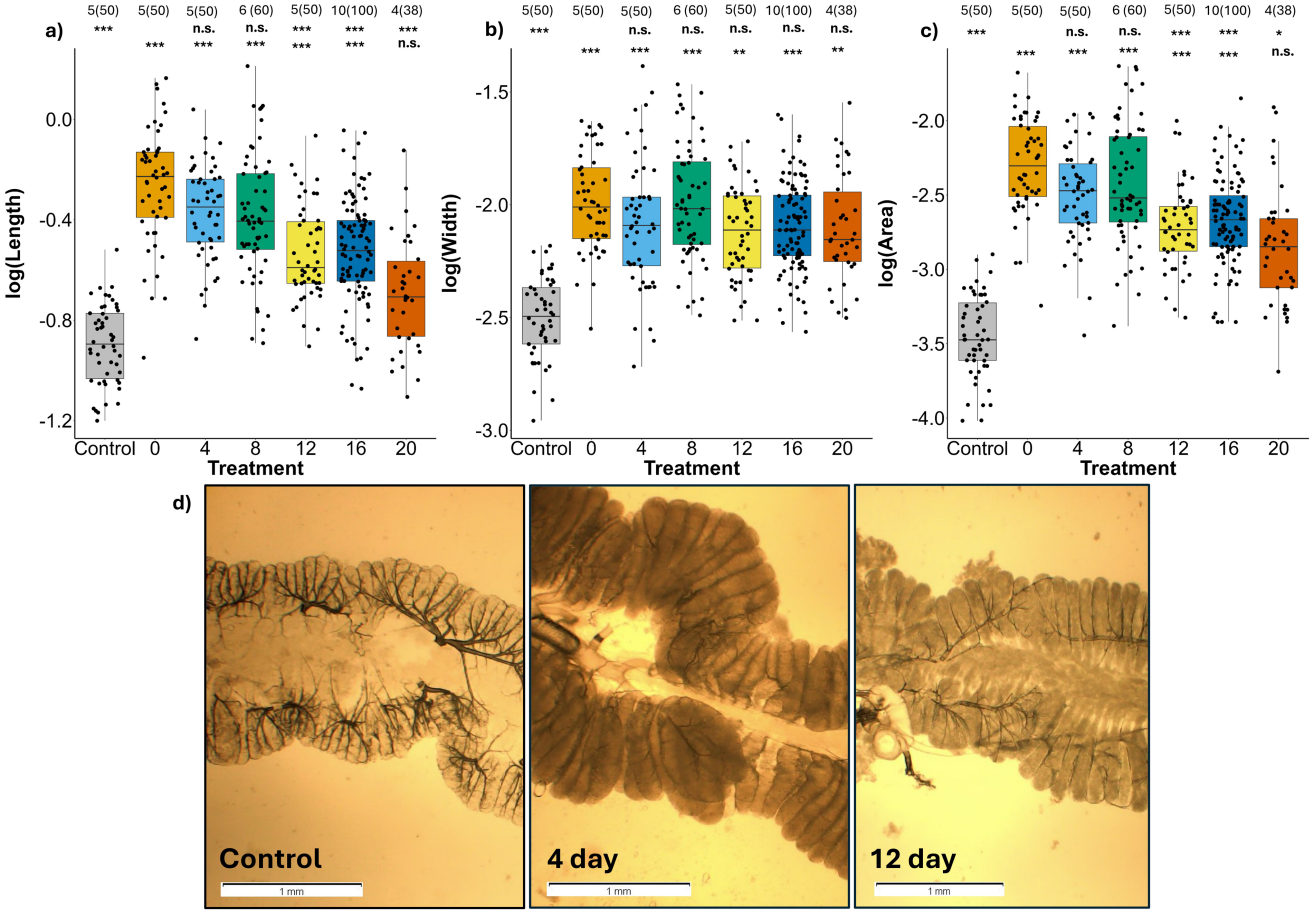
Size of adult *L. zonatus* crypts **(a)** length and **(b)** width in millimeters, and **(c)** total area in mm^2^ relative to treatment. **(d)** Example of M4 crypts of adults. Left: an aposymbiotic negative control. Middle: an adult fed *Caballeronia* at 4 days after reaching the 2nd instar. Right: an adult fed *Caballeronia* 12 days after reaching the 2nd instar. The numbers above the bars are the number of insects sampled and within parentheses is the total number of crypts measured. Scale bars represent 1 mm. The upper row of asterisks represents the *p* values when each treatment was compared with the 0-day treatment (0), the lower row represents comparisons with the negative control (Control) (n.s. = non-significant, * ≤ 0.05, ** ≤ 0.01, *** ≤ 0.001).

Delay before symbiont acquisition significantly reduced the ability of the M4 crypts to grow to accommodate the bacterial population. Midgut 4 crypts varied significantly among treatments by length (LRT = 58.55, *p =* 8.86 × 10^−11^), width (LRT = 33.10, *p =* 1.01 × 10^−5^), and total area (LRT = 59.13, *p* = 6.76 × 10^−11^). The length of the M4 crypts appeared most affected by delay (Figure 6a). Total crypt length and total crypt area declined significantly beginning in the 12-day treatment (Length: *t* = −3.81, *p* = 0.003; Area: *t =* −3.28, *p* = 0.013) with all subsequent treatments varying significantly compared with the 0-day treatment (Crypt Length: Negative Control: *t =* −8.94, 1.51 × 10^−9^; 4 day: *t* = −1.51, *p* = 0.468; 8 day: *t =* −1.85, *p* = 0.287; 16 day: *t =* −4.25, *p* = 9.16 × 10^−4^; 20 day: *t =* −5.70, *p* = 1.27 × 10^−5^; Crypt Area: Negative Control: *t* = −9.18, *p* = 8.02 × 10^−10^; 4 day: *t* = −1.56, *p* = 0.44; 8 day: *t* = −1.01, *p* = 0.77, 16 day: *t =* −3.39, *p* = 0.010; 20 day: *t* = −4.15, *p* = 0.001) (Supplementary Tables 13, 17). For crypt width, only the negative control varied significantly from the 0 day treatment (*t =* −5.44, *p* = 2.91 × 10^−5^, 4 day: *t* = −0.93, *p* = 0.81; 8 day: *t* = 0.05, *p* = 1.00; 12 day: *t* = −1.36, *p* = 0.558; 16 day: *t =* −1.25, *p* = 0.63; 20 day: *t* = −1.09, *p* = 0.72) (Supplementary Table 15). When compared with the negative control, crypt width and area were significantly different from all treatments (Supplementary Tables 16, 18). For crypt length, all treatments were significantly different from the negative control except for the 20 day treatment, although crypt lengths in this treatment approached statistical significance (*t* = 2.65, *p* = 0.054) (Supplementary Table 14).

## Discussion

### Delay is costly

Our results confirmed the critical nutritional role of *Caballeronia* for *L. zonatus* and suggested that delaying symbiont acquisition was costly to the bug host. We found that individuals that experienced greater delays experienced greater costs. When *Caballeronia* was not ingested within ~ 8 days of development to the 2nd instar, there was a significant increase in development time, juvenile mortality, and a decrease in adult mass. While symbiont acquisition did occur after this interval, bugs were less likely to be colonized by the bacteria and their symbiotic organ was less developed than those bugs that were colonized at earlier stages. As delay increased, the consequences appeared to compound, beginning in the 16 day treatment with individuals being comparable to those in the negative control. The consequences of being aposymbiotic were severe. Fewer than one third of aposymbiotic *L. zonatus* survived to adulthood and those survivors had an almost 60% longer development time and were ~40% the mass of symbiotic adults.

Longer development times alone can have significant consequences for insect survival. The juvenile stages are often the most vulnerable to attack from natural enemies and susceptible to harsh environmental conditions (Benk et al., 2020), due in part to reduced mobility as compared to adults. Juvenile insects are limited in their ability to disperse to escape natural enemies, acquire resources, or to behaviorally regulate body temperature. Congruent with the “slow growth—high mortality” hypothesis (Feeny, 1976; Clancy and Price, 1987), an increase in an organism’s developmental period or “window of vulnerability” (Benrey and Denno, 1997) is also likely to increase mortality.

For those *L. zonatus* that survived to adulthood, we observed that delays beyond 12 days before symbiont acquisition resulted in significantly decreased body size, and by 16 days delay, body size was not significantly different from the negative control. We might expect that even slight decreases in body size in bugs that eventually acquire the symbiont will reduce reproductive success. Insect reproductive success is highly correlated with adult body size in both males and females (Kingsolver and Huey, 2008), with smaller females being generally less fecund (Honěk, 1993). At low population densities, smaller size may incur even greater costs because mate-finding may require a larger investment to physically locate mates (e.g., Kuussaari et al., 1998) and reduce time spent feeding and growing. *Leptoglossus zonatus,* like other coreids, often has several overlapping generations per year (Daane et al., 2016; Daane et al., 2019). This “generational smearing” (Bjørnstad et al., 2016) is driven by multiple factors, including temperature and microclimate, that can modulate development at the individual level (Bjørnstad et al., 2016). In the context of the observed results, delays in symbiont availability will influence development time and developmental synchrony of cohorts. Variability within a cohort may be of little consequence in a large population where individuals would be able to mate with members of previous or following generations. In a highly variable environment, a staggered microbe-mediated emergence could even be considered beneficial bet-hedging if it spread exposure risk to environmental hazards or reduced competition (Metcalf et al., 2019). At low densities or in newly invaded areas, however, individuals with delayed development might emerge to find few mating opportunities.

We found that a delay of a week or more before symbiont acquisition may be highly costly to bugs. If acquisition is delayed long enough, the cost is increased and nymphs might fail to acquire *Caballeronia* at all, and will produce no offspring (Umanzor et al., 2025). After 12 days of reaching the second instar, we observed a precipitous decline in symbiont acquisition success, from >90% of bugs that acquired *Caballeronia* if the delay was within 8 days, decreasing to 75% success after 12 days, falling to 50% at 16 days, to 12% at 20 days and 0% at 24 days. The start of the decline of acquisition coincided with nymphal instar; at 12 days, aposymbiotic bugs began to molt to the 4th instar, and by 16 days ~90% had reached this stage (Figure 4b). The exact cause of this failure to acquire the symbiont is unclear, though these findings reflect a similar pattern observed in *R. pedestris* (Kikuchi et al., 2011b). In *R. pedestris* it has been documented that the CR closes when the midgut crypts are colonized (Kikuchi et al., 2020). It may be that while *Caballeronia* colonization causes the closing of the CR, closing may occur regardless in aposymbiotic bugs that have reached later instars. The mechanism that prevents late colonization of the midgut is unknown but could be related to our observation of limits to plasticity in gut morphogenesis of bugs in response to the symbiont after delays in acquisition.

Regardless of the mechanism, in the current study, a decline in acquisition success with increasing delay (Figure 1) was an important component in the decline of all other measures of bug performance. While ideally it might be interesting to compare the fitness of *Caballeronia* colonized and uncolonized individuals with increasing delay, the precipitous decline in acquisition rates of later treatments confounds all the subsequent fitness measures. With every comparison against the aposymbiotic negative control, however, it is apparent that whether acquisition was successful or not, delays of between 12 and 16 days or longer result in host individuals with fitness as severely compromised as ones that never find the symbiont at all (Figures 2, 3, 5).

Delay also influenced gut morphogenesis. We found that surviving adults that experienced acquisition delay displayed reduced expansion of the M4 crypts after colonization. Jang et al. (2023) showed colonization of the M4 *Riptortis pedestris* symbiotic organ by *Caballeronia* triggers growth of the midgut crypts to develop and elongate. Similarly, we saw an enlargement of the M4 in *L. zonatus* adults for all treatments that were colonized by *Caballeronia*, relative to aposymbiotic bugs. However, our data suggests that this developmental response to the symbiont is time-limited. After a delay of 12 days from the 2nd instar, crypt lengths in adults were significantly reduced, even if still significantly longer than in individuals never fed *Caballeronia,* suggesting reduced developmental plasticity at this stage. It is possible that as *L. zonatus* nymphs develop, the gut becomes less receptive to the presence of the symbiont, and both passage through the CR and the triggering of crypt development become more uncertain. Furthermore, the reduced crypt size may house fewer bacteria, and if so lower bacterial titer is likely to reduce the nutritional benefit to the host, although the exact relationship of symbiont titer and benefit is unclear. Taken together, these results illustrate a narrow interval of just over a week where symbiont acquisition can occur before bug survival and performance is compromised.

A possible caveat to our observation of reduced M4 crypt size in later delay treatments could be that nymphs simply had fewer days for the gut to respond to the symbiont before eclosion to adulthood, when presumably development stops. To address this possibility, we conducted an analysis of the days remaining to adulthood following symbiont inoculation. Indeed, bugs in later delay treatment groups did have significantly less time between symbiont acquisition and adult eclosion than did those in earlier delay treatments (see Supplementary methods, Supplementary Figure 1; Supplementary Tables 13, 14). However, even in the 20 day treatment, bugs required on average 16 ± 4.78 days before eclosion to adulthood (Supplementary Figure 1; Supplementary Table 14). This relatively long period is because nymphs waiting for their symbiont are developmentally delayed, with aposymbiotic bugs requiring twice as long to reach adulthood compared to symbiotic individuals (Figure 4b) (Hunter et al., 2022). Due to the long period of time required to develop to adulthood in every delay treatment, the time available for gut morphogenesis would appear to be more than sufficient. Jang and Kikuchi (2020) found that in *R. pedestris*, the size of both crypts and M4 became significantly larger in just 3 days following symbiont establishment (Jang and Kikuchi, 2020). Therefore, in our system, the number of days remaining to reach adulthood is unlikely to be limited or to curtail morphogenesis, the onset of which is observable within a matter of hours.

### Development and morphogenesis are hampered by acquisition delay

Our finding that, like *R. pedestris, L. zonatus* gut crypts develop in response to the presence of *Caballeronia* (Jang et al., 2023) is an example of developmental symbiosis, a term coined for the ontogeny and morphogenesis of tissues induced by microbial symbionts. This area has received significant attention in the last decade (Gilbert et al., 2015; Carrier and Bosch, 2022). The presence of microbes has been observed to induce tissue development, including oogenesis (Dedeine et al., 2001), gut tissue morphogenesis (Kikuchi et al., 2020; Jang et al., 2023), the establishment of gut peristalsis in hydra (Murillo-Rincon et al., 2017) and larval settlement in marine species (Freckelton et al., 2017). It is not clear, however, if the time or developmental stage window for potential response of the host tissue to the microbes that we observed in the current study is common in other systems. For *L. zonatus*, one could speculate that important developmental programs associated with older symbiotic instars may trade-off against the phenotypic plasticity necessary for M4 colonization. Some of these programs may proceed even in the absence of the symbiont, including the potential closure or obstruction of the constricted region, (Kikuchi et al., 2011b; Ohbayashi et al., 2015). In general, when the symbiont or microbial community is omnipresent, the question may not be relevant. But, for specialized symbiont-host interactions, a heterogeneous environment may make any potential time limit for a morphological response risky.

### Life history implications

Previous research has shown that *Caballeronia* may be scarce in the tree canopy (Sullivan et al., 2025). For *L. zonatus* nymphs, just 3–4 mm in length, this means a meters-long journey to the soil or other potential sources of *Caballeronia* with no guarantees of successful acquisition. Such a journey is likely to involve significant risk. Leaving the “natal herd” alone is likely to increase risk of predation and desiccation (Evans and Schmidt, 1990). The danger is further compounded when one considers the energetic demands of dispersal and return to the canopy where food resources are located, demands which would be accentuated in an aposymbiotic bug. However, failing to acquire the symbiont is also lethal in this species. The lack of a symbiont likely drives dispersal, as deprivation of one’s nutritional symbiont has been shown to increase movement in juvenile of the stink bug, *Megacopta punctatissima* (Hosokawa et al., 2008) as well as in the squash bug, *Anasa tristis* (Villa et al., 2023) suggesting the increased movement may increase the likelihood of symbiont acquisition. We have observed similar behavior in *L. zonatus* under laboratory conditions: nymphs are highly active prior to symbiont feeding and may become largely sessile once the symbiont is ingested (L. Sullivan, unpublished data). Given potential predation, desiccation and starvation risks of dispersal to find the symbiont, the window for acquiring the symbiont is likely to be even narrower than we observed in our laboratory study where nymphs are protected and fed. The rarity of *Caballeronia* in the tree canopy (Sullivan et al., 2025) and the energetic investment required to locate *Caballeronia* suggests that even a few days of delay could be costly to a bug’s performance.

## Conclusion

It is not clear how frequently there is a delay in *L. zonatus* symbiont acquisition in nature or how often this arboreal bug fails to find *Caballeronia* when nymphs are not confined to the canopy. It may be that restricted symbiont access as a phenomenon is rare in nature, or that finding the symbiont is simply one more hazard for herbivores that regularly face a myriad of obstacles including low food quality, weather events, and natural enemies (Cornell and Hawkins, 1995). Recently it has been shown has shown that a congener, *L. phyllopus,* may enrich the soil population with excreted *Caballeronia* (Parajuli et al., 2025). The roles of the dispersion of the *Caballeronia* in the environment as well as of environmental enrichment of *Caballeronia* from bug populations in increasing the probability of attraction and success of future generations of *L. zonatus* would be interesting topics for future study.

## Data Availability

The raw data supporting the conclusions of this article will be made available by the authors, without undue reservation.
