# Supplementary material for: Late is not great: fitness implications of delayed symbiont acquisition
Source: Front Microbiol. 2026 May 14;17:1786420. doi: 10.3389/fmicb.2026.1786420 (PMC13216211; doi:10.3389/fmicb.2026.1786420)
Supplement: Supplementary file 1 [file Data_Sheet_1.docx]

Supplementary Material

# Supplementary Figures and Tables

SI Table 1. Contrasts for symbiont acquisition. All treatments are compared to the 0 day positive control. Negative control treatments never received *Caballeronia* and were therefore excluded from the analysis.


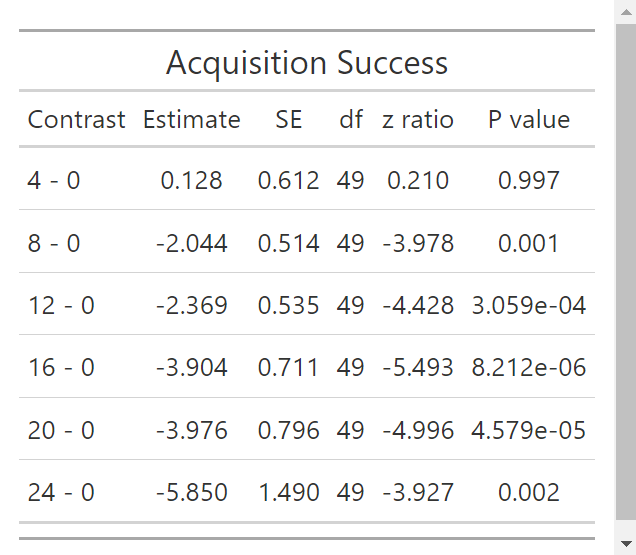


SI Table 2. Summary table for symbiont acquisition. N is the total number of replicates within each treatment.


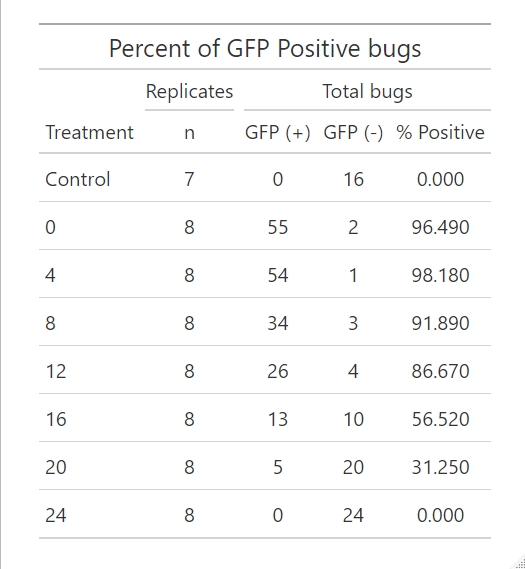


SI Table 3. Summary table for survivorship. N is the total number of replicates within each treatment. Number of adults is the number of surviving bugs.


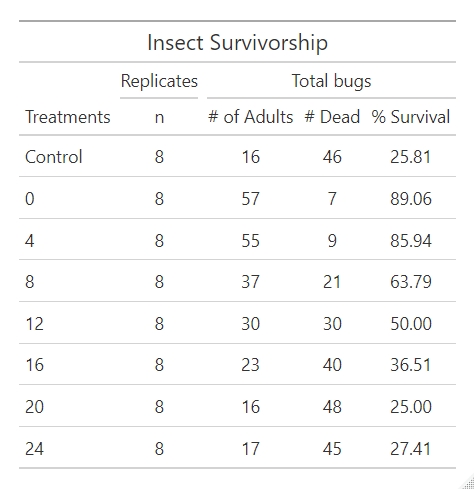


SI Table 4. Contrasts for development time. All treatments are compared to the 0 day treatment.


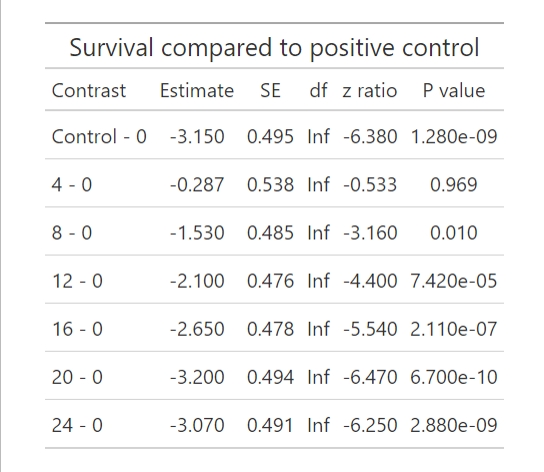


SI Table 5. Contrasts for survivorship. All treatments are compared to the negative control.


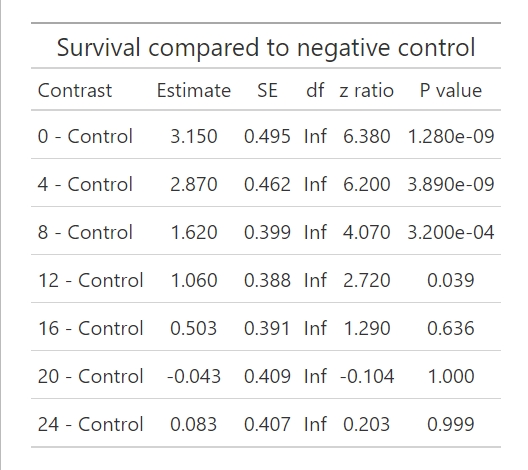


SI Table 6 1. Mean days to adulthood by treatment

##
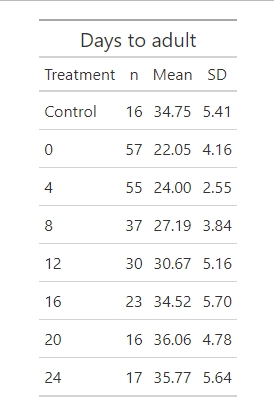


SI Table 7. Contrasts for development time. All treatments are compared to the 0 day treatment.


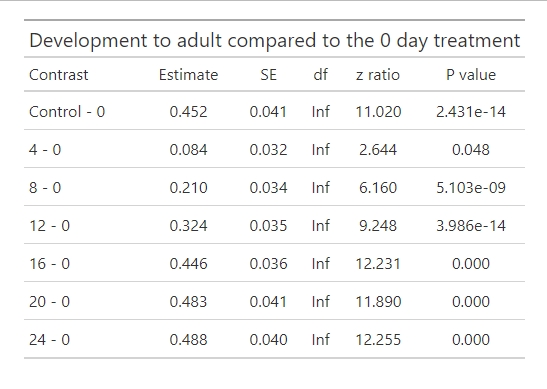


SI Table 8. Contrasts for development time. All treatments are compared to the negative control.


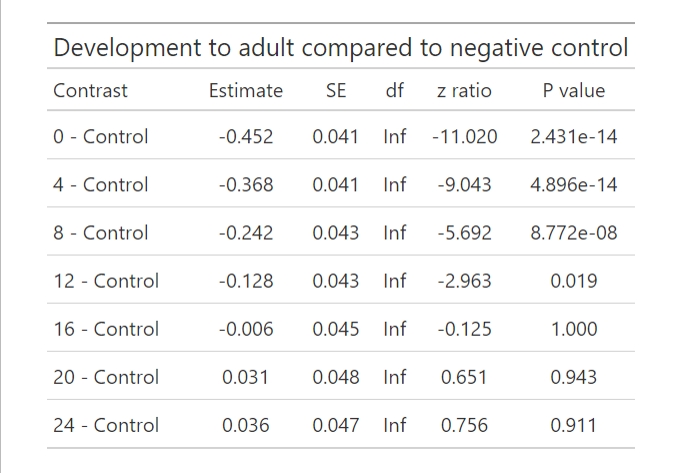


SI Table 9. Mean adult mass from each treatment group.


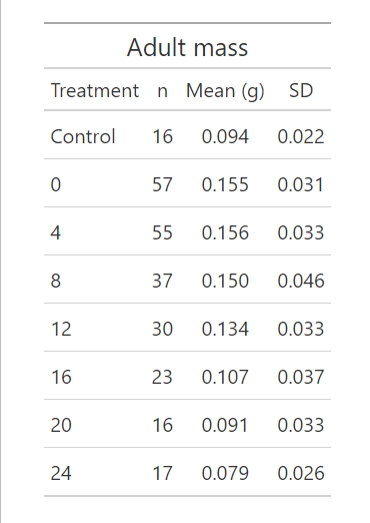


SI Table 10. Contrasts for adult mass. All treatments are compared to the 0 day treatment.


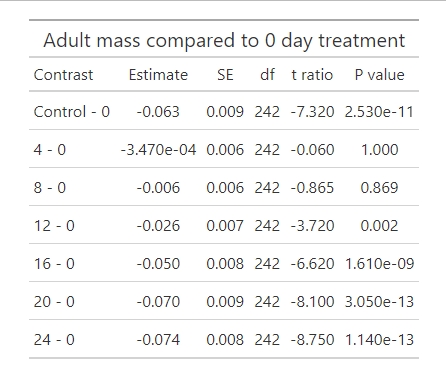


SI Table 11. Contrasts for adult mass. All treatments are compared to the negative control.


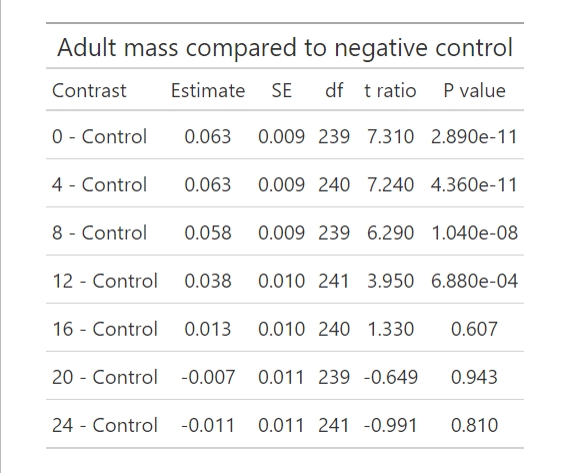


SI table 12. Summary table of crypt measurements


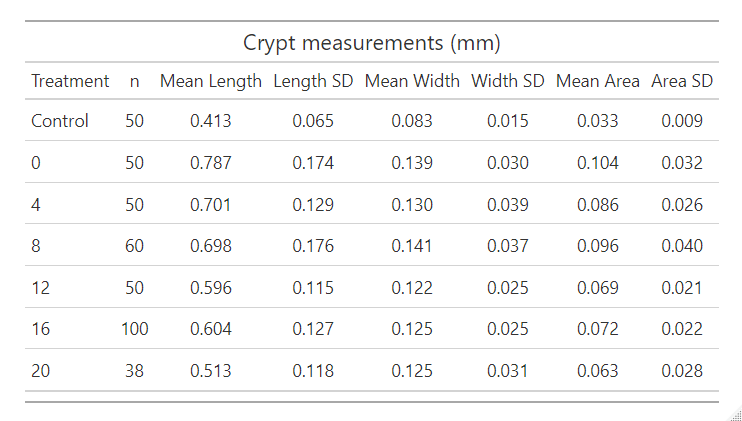


SI table 13. Contrasts of crypt lengths. All treatments are compared to the 0 day treatment.


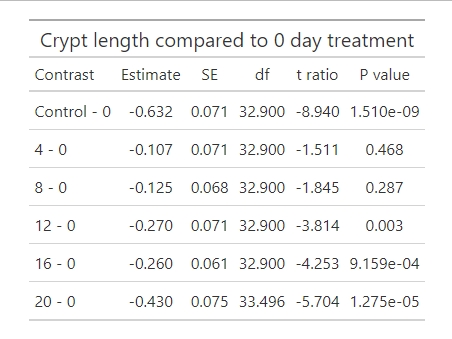


SI table 14. Contrasts of crypt lengths. All treatments are compared to the negative control.


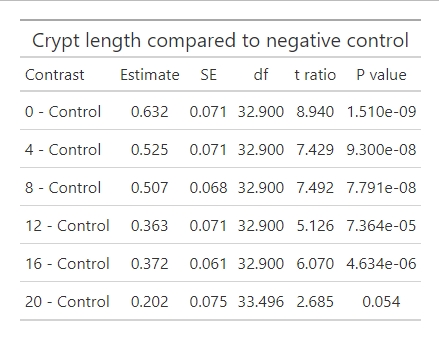


SI table 15. Contrasts of crypt widths. All treatments are compared to the 0 day treatment.


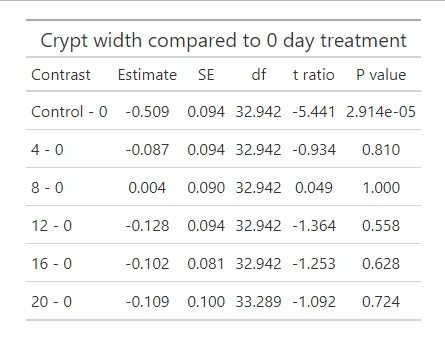


SI table 16. Contrasts of crypt widths. All treatments are compared to the negative control.


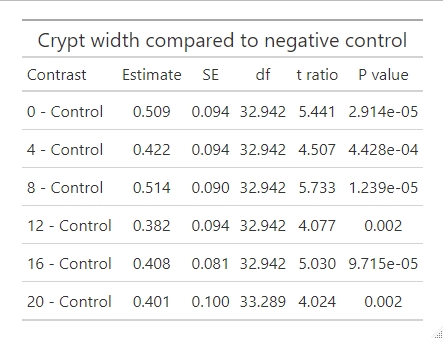


SI table 17. Contrasts of crypt areas. All treatments are compared to the 0 day treatment.


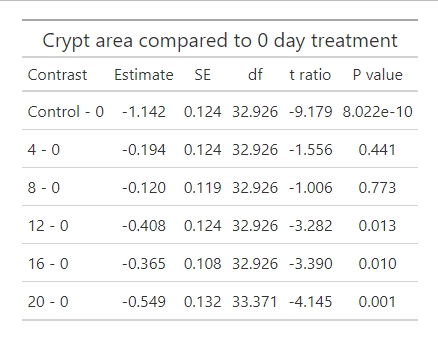


SI table 18. Contrasts of crypt areas. All treatments are compared to the negative control.


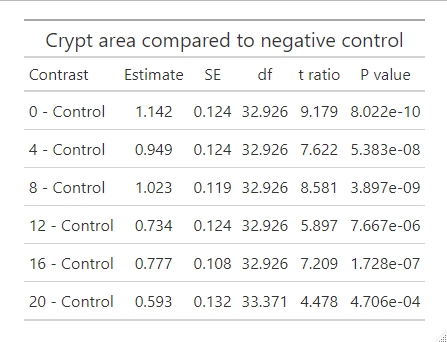


**2 Supplemental Methods and Results**

**2.1 Development from symbiont acquisition to adulthood**.

Development time from acquisition was calculated by subtracting the eclosion date for the insect from the date when they were fed *Caballeronia*. The 24 day treatment was excluded from the analysis as no bugs in this treatment were found to be colonized by GFP-*Caballeronia.* To estimate the number of days from symbiont feeding to adulthood across treatments and to determine whether treatments varied in this measure, we compared development to adulthood from feeding date in days across treatments using a generalized linear regression with a quasipoisson estimation. Days to adulthood was the response variable, with treatment as the explanatory variable. We found time from symbiont acquisition to eclosion was impacted significantly by treatment (*F* = 7.89, *P* = 7.79x10^-7^). Pairwise comparisons with the 0d control can be found in SI table 19 and SI Figure 1, with summary statistics present in SI table 20.

SI Table 19. Comparison of development from inoculation date to adulthood. Contrasts represent comparison with the 0 day treatment.


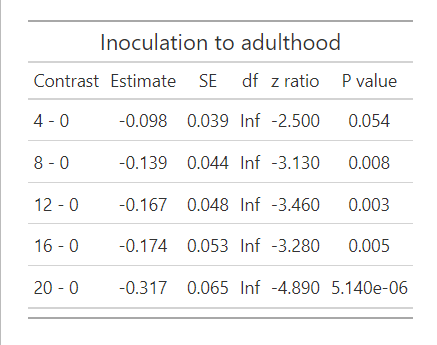


SI Table 20. Summary table for development time to adulthood from inoculation.


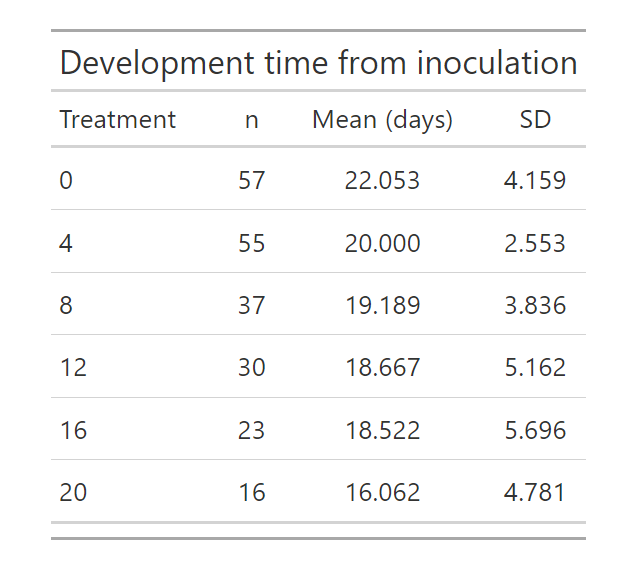


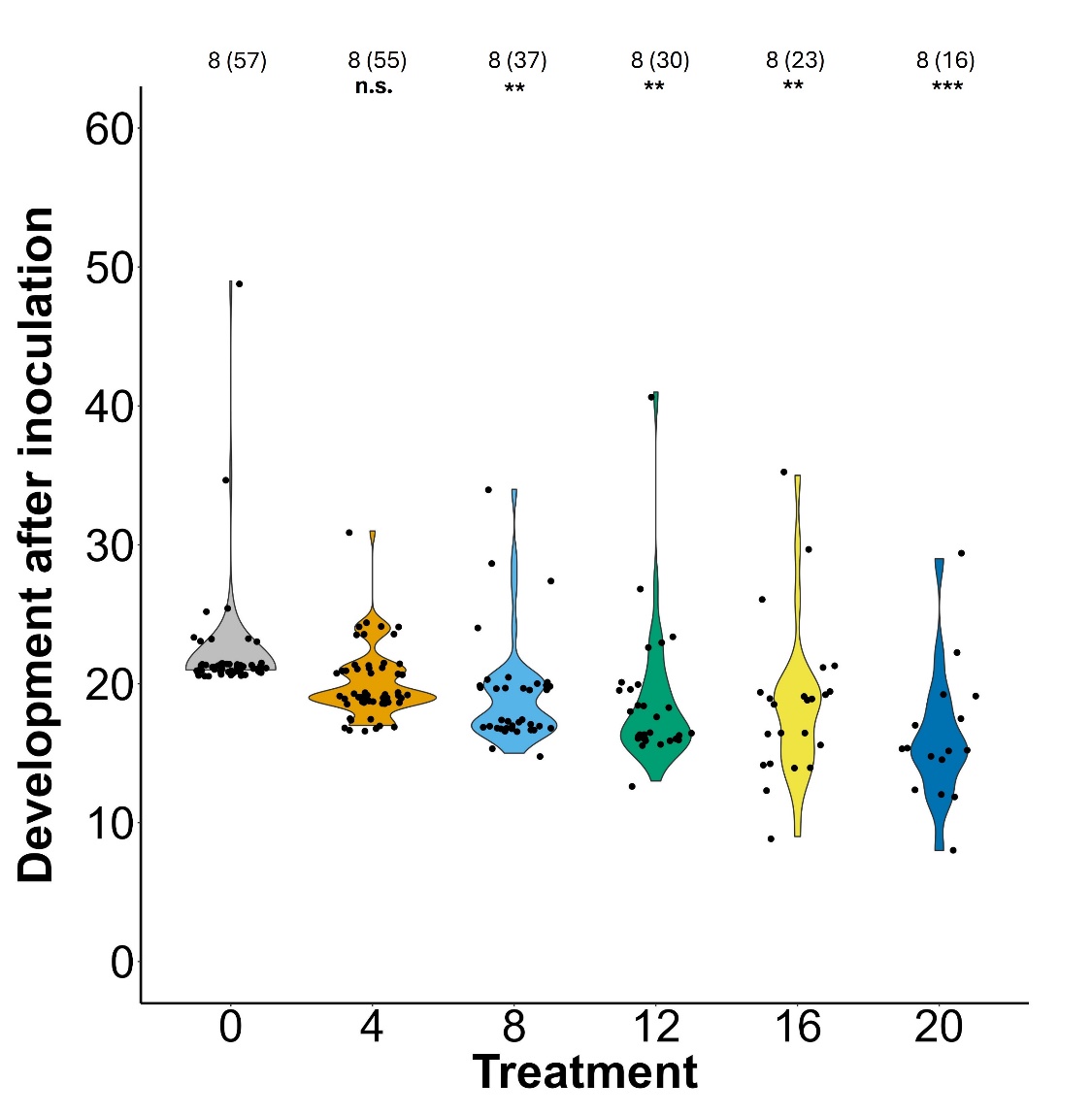


**Supplementary figure 1**. Development time to adulthood from date of inoculation by treatment. Asterisks represent the P values when each treatment was compared with the 0 day treatment (0; n.s. = non-significant, * ≤ 0.05, ** ≤ 0.01, *** ≤ 0.001).
